# A novel maize microRNA negatively regulates resistance to *Fusarium verticillioides*

**DOI:** 10.1111/mpp.13240

**Published:** 2022-06-14

**Authors:** Yufang Xu, Renjie Wang, Peipei Ma, Jiansheng Cao, Yan Cao, Zijian Zhou, Tao Li, Jianyu Wu, Huiyong Zhang

**Affiliations:** ^1^ College of Life Sciences Henan Agricultural University Zhengzhou China; ^2^ State Key Laboratory of Wheat and Maize Crop Science, Collaborative Innovation Center of Henan Grain Crops Henan Agricultural University Zhengzhou China

**Keywords:** disease resistance, *Fusarium verticillioides*, gibberellin, microRNA, zma‐unmiR4

## Abstract

Although microRNAs (miRNAs) regulate the defence response against multiple pathogenic fungi in diverse plant species, few efforts have been devoted to deciphering the involvement of miRNA in resistance to *Fusarium verticillioides*, a major pathogenic fungus affecting maize production. In this study, we discovered a novel *F. verticillioides‐*responsive miRNA designated zma‐unmiR4 in maize kernels. The expression of zma‐unmiR4 was significantly repressed in the resistant maize line but induced in the susceptible lines upon exposure to *F. verticillioides* exposure, whereas its target gene *ZmGA2ox4* exhibited the opposite pattern of expression. Heterologous overexpression of *zma‐unmiR4* in *Arabidopsis* resulted in enhanced growth and compromised resistance to *F. verticillioides*. By contrast, transgenic plants overexpressing *ZmGA2ox4* or the homologue *AtGA2ox7* showed impaired growth and enhanced resistance to *F. verticillioides*. Moreover, zma‐unmiR4‐mediated suppression of *AtGA2ox7* disturbed the accumulation of bioactive gibberellin (GA) in transgenic plants and perturbed the expression of a set of defence‐related genes in response to *F. verticillioides*. Exogenous application of GA or a GA biosynthesis inhibitor modulated *F. verticillioides* resistance in different plants*.* Taken together, our results suggest that the zma‐unmiR4–*ZmGA2ox4* module might act as a major player in balancing growth and resistance to *F. verticillioides* in maize.

## Graphical abstract

Maize zma‐unmiR4 functions as a crucial modulator to regulate resistance to *Fusarium verticillioides* and plant growth by mediating gibberellin accumulation.

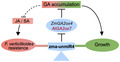

## 1 INTRODUCTION

MicroRNAs (miRNAs), a class of 20–24‐nucleotide endogenous noncoding RNAs, have been widely found in eukaryotes and act as gene repressors by directing the cleavage or translational repression of the target transcripts (Voinnet, 2009). It has been well documented that miRNAs are involved in the control of some of the most challenging plant traits in agricultural production, such as plant development and architecture, and environmental stress and defence responses (Kumar, 2014; Rubio‐Somoza & Weigel, 2011; Sunkar et al., 2012).

Accumulating evidence indicates that miRNAs regulate multiple biotic stress responses in plants, including interactions with fungi (Chen et al., 2021; Hu et al., 2020; Zhang et al., 2016), viruses (Mengistu & Tenkegna, 2021; Yang et al., 2016; Yao et al., 2019), bacteria (Liu et al., 2019; Navarro et al., 2006; Zhang et al., 2011), and insects (Feng et al., 2021; Li et al., 2018). The expression of miR393, the first miRNA identified to be involved in plant immunity, is induced by bacterial flagellin‐derived peptide and restricts the growth of *Pseudomonas syringae* by repressing auxin signalling (Navarro et al., 2006). miR159a plays a positive role in rice resistance to *Magnaporthe oryzae* (Chen et al., 2021), whereas miR156 negatively regulates rice resistance to bacterial blight by *Xanthomonas oryzae* (Liu et al., 2019). In addition, the important roles of miR160a (Li et al., 2014), miR166 (Zhang et al., 2016), miR528 (Wu et al., 2017), miR398b (Li et al., 2014), miR164 (Hu et al., 2020), and miR168 (Wu et al., 2015) in disease resistance by regulating specific target genes in various crops have been well characterized. For instance, miR528 negatively regulates rice resistance to rice stripe virus by cleaving the transcripts of the *l‐ascorbate oxidase* (*AO*) gene (Wu et al., 2017). Loss of function of the Osa‐miR159a target genes, including *OsGAMYB*, *OsGAMYBL*, and *OsZF*, results in enhanced resistance to *M. oryzae*, consistent with the related phenotypes of Osa‐miR159a overaccumulation plants (Chen et al., 2021). miR156 negatively regulates rice resistance against bacterial blight through decreasing the expression levels of its targets *IPA1* and *OsSPL7* (Liu et al., 2019). Considering the extensive regulation of miRNAs during plant immunity, further characterization of pathogen‐responsive miRNAs and resultant miRNA‐mediated disease defence processes will have a profound impact on the development of new strategies for controlling disease damage in crop production.

*Fusarium verticillioides* is one of the most commonly occurring pathogenic fungi and causes various prevalent diseases in crops, especially maize, posing a great challenge to food and feed safety (Gai et al., 2018; Ju et al., 2017; Liu et al., 2020; Mu et al., 2018; Septiani et al., 2019). *F. verticillioides* infection occurs throughout the whole growth period of maize and results in seedling blight, stalk rot, ear rot, and seed rot (Machado et al., 2013; Septiani et al., 2019; Stagnati et al., 2019). Most importantly, *F. verticillioides*‐infected plants or seeds may accumulate fumonisins, a family of mycotoxins associated with several diseases in livestock and humans and classified as probable carcinogens (Rosa Junior et al., 2019). Thus, it is of great significance to dissect the molecular mechanism of resistance to *F. verticillioides*. Although many genetic and omics studies have identified a series of quantitative trait loci/genes associated with *F. verticillioides* resistance (Butrón et al., 2019; Chen et al., 2016; Lanubile et al., 2017; Maschietto et al., 2017; Schiwek et al., 2020; Yao et al., 2020), the molecular mechanisms underlying the response of plants to *F. verticillioides* remain largely elusive, especially the role of miRNAs in this process. In our previous study using high‐throughput sequencing (Zhou et al., 2020), a number of miRNAs, including known and new predicted miRNAs, were identified to be potentially associated with resistance to *F. verticillioides* ear rot. Further functional analysis of these miRNAs is important to dissect the molecular mechanisms underlying the plant–*F. verticillioides* interaction and ultimately improve disease resistance.

In the current study, we focused on a novel *F. verticillioides‐*responsive miRNA designated zma‐unmiR4 and aimed to reveal its function in the response of plants against *F. verticillioides*. We found that the expression levels of zma‐unmiR4 were significantly down‐regulated in the resistant maize line but up‐regulated in the susceptible lines after *F. verticillioides* infection, whereas the target gene *ZmGA2ox4* displayed the opposite profiles of expression. Heterologous accumulation of zma‐unmiR4 resulted in impaired resistance to *F. verticillioides* infection and enhanced growth in *Arabidopsis*; however, transgenic plants overexpressing *ZmGA2ox4* or the homologue *AtGA2ox7* showed high resistance to *F. verticillioides* as well as retarded growth. Further analyses indicated that zma‐unmiR4 was able to regulate *F. verticillioides* resistance through gibberellin (GA) signalling by suppressing *AtGA2ox7* expression in *Arabidopsis*. These results provide direct evidence for the crucial role of zma‐unmiR4 in regulating plant growth and disease resistance to *F. verticillioides*.

## 2 RESULTS

### 2.1 zma‐unmiR4 is a novel maize miRNA responsive to *F. verticillioides* infection

**FIGURE 1:**
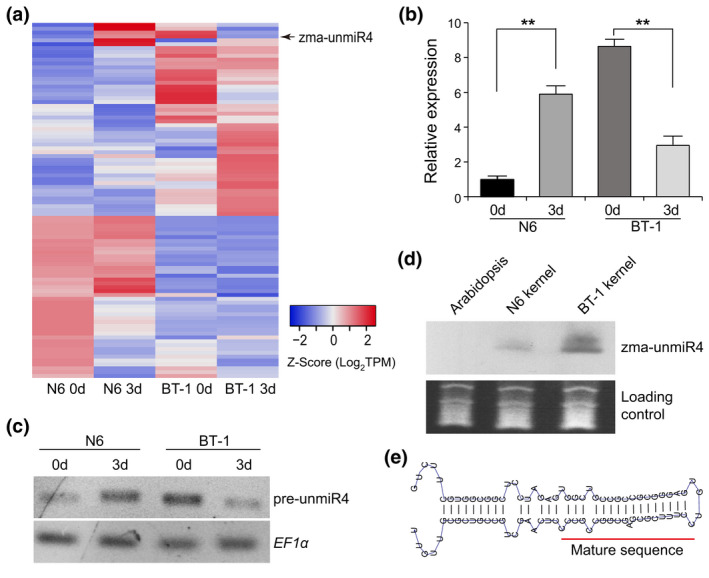
Identification and validation of zma‐unmiR4. (a) Expression heatmap of the 92 predicted novel miRNA candidates identified by small RNA sequencing in BT‐1 and N6 kernels after *Fusarium verticillioides* inoculation (Zhou et al., 2020). Maize kernels of BT‐1 and N6 at 0 or 3 days postinoculation were sampled for construction of a small RNA library. (b) Reverse transcription (RT)‐quantitative PCR analysis of differential expression of zma‐unmiR4 in BT‐1 and N6 maize lines after *F. verticillioides* inoculation. Maize *EF1a* was used as the internal control. Data are means ± standard deviation from three biological replicates. **p < 0.01 by Student's *t* test. (c and d) Verification of zma‐unmiR4 production by RT‐PCR amplifying its precursor (c) and RNA blotting (d). (e) Hairpin structure of zma‐unmiR4 predicted by RNAfold software.

Deep sequencing of small RNA libraries from maize kernels untreated or treated with *F. verticillioides* previously revealed a number of *F. verticillioides*‐responsive miRNAs (Zhou et al., 2020), including 92 potentially novel miRNAs. These predicted miRNAs displayed various expression profiles in response to *F. verticillioides* (Figure 1a). One novel miRNA candidate, designated zma‐unmiR4, was characterized in more detail for its differential expression in the *F. verticillioides*‐susceptible maize line N6 and the resistant line BT‐1 (Figure 1b). Amplification of its precursor sequence indicated that zma‐unmiR4 is transcribed as an individual transcriptional unit from the maize genome (Figure 1c). In addition, zma‐unmiR4 transcription was confirmed through RNA blotting in maize kernels (Figure 1d). A high degree of complementarity for the precursor structure was observed using the RNAfold web server (Figure 1e). These observations support the notion that zma‐unmiR4 represents a novel miRNA potentially regulating resistance to *F. verticillioides* in maize. Moreover, zma‐unmiR4 was found to be expressed in various maize tissues (Figure S1), implying its potential functions during various developmental stages.

### 2.2 *ZmGA2ox4* and its homologue *AtGA2ox7* are the targets of zma‐unmiR4

**FIGURE 2:**
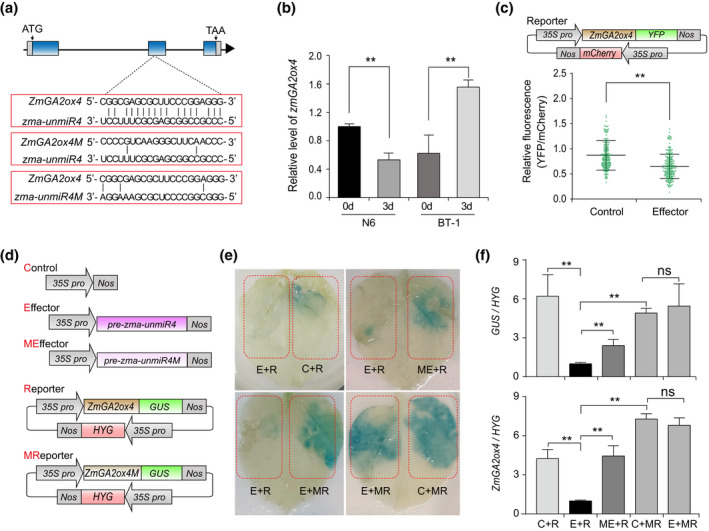
Maize *ZmGA2ox4* is targeted by zma‐unmiR4. (a) Schematic diagram of *ZmGA2ox4* showing the target site of zma‐unmiR4, the mutated target site of *ZmGA2ox4* (*ZmGA2ox4M*), and mutated zma‐unmiR4 (*zma‐unmiR4M*). (b) *ZmGA2ox4* was differentially regulated in BT‐1 and N6 kernels after *Fusarium verticillioides* inoculation. Maize *EF1a* was used as internal control. (c) Maize protoplasts were cotransfected with the reporter plasmid and empty vector or the *35S:pre‐unmiR4* effector. The fluorescence intensity of yellow fluorescent protein (YFP) was normalized to mCherry. Data are represented as mean ± standard deviation (*n* ≥ 200 cells). (d) Structure of various constructs used in the transient transformation assay in tobacco plants. (e and f) Tobacco leaves were cotransfected with different reporter plasmids and different effector plasmids. After 2 days, the transfected leaves were used for β‐glucuronidase (GUS) staining (e) and isolation of total RNA for reverse transcription‐quantitative PCR analysis to determine the expression levels of *GUS* and *ZmGA2ox4* (f). C, E, ME, R, and MR indicate various constructs of control, effector, mutated effector, reporter, and mutated reporter in panel (d), respectively. Data are means ± standard deviation from three biological replicates. **p < 0.01 by Student's *t* test.

Based on target gene prediction (http://rna.informatik.uni‐freiburg.de), zma‐unmiR4 showed extensive sequence complementarity with the gene *Zm00001d017294* encoding gibberellin 2‐oxidase 4 (*ZmGA2ox4*; Figure 2a). Notably, the accumulation of *ZmGA2ox4* transcripts was drastically increased in BT‐1 but decreased in N6 after *F. verticillioides* inoculation (Figure 2b), in contrast to zma‐unmiR4 expression levels (Figure 1a–c). To confirm that *ZmGA2ox4* is regulated by zma‐unmiR4 in planta, the constructs expressing *ZmGA2ox4‐YFP* (*35S:ZmGA2ox4‐YFP*) and zma‐unmiR4 (*35S:pre‐unmiR4*) were cotransformed into maize protoplasts (Figure 2c,d), and yellow frluorescent protein (YFP) signals were seen to be significantly decreased in the protoplasts (Figure 2c). We then expressed *35S:ZmGA2ox4‐GUS* and *35S:pre‐unmiR4* transgenes in tobacco leaves, and found that the β‐glucuronidase (GUS) signals were nearly undetectable compared with the strong GUS staining when expressing *35S:ZmGA2ox4‐GUS* alone (Figure 2d,e). Reverse transcription‐quantitative PCR (RT‐qPCR) also showed that *ZmGA2ox4* transcript levels were significantly decreased when both transgenes were coexpressed (Figure 2f). To confirm a direct interaction between zma‐unmiR4 and *ZmGA2ox4*, we also constructed a reporting system containing mutated vectors of *ZmGA2ox4* (*ZmGA2ox4M*) and zma‐unmiR4 (*zma‐unmiR4M*) (Figure 2a,d). As shown in Figure 2e,f, neither cotransformation of *zma‐unmiR4M* and normal *ZmGA2ox4* nor cotransformation of *ZmGA2ox4M* and normal zma‐unmiR4 effectively reduced the GUS signals and *ZmGA2ox4* expression. Together, these results demonstrate that *ZmGA2ox4* is a target of zma‐unmiR4.

**FIGURE 3:**
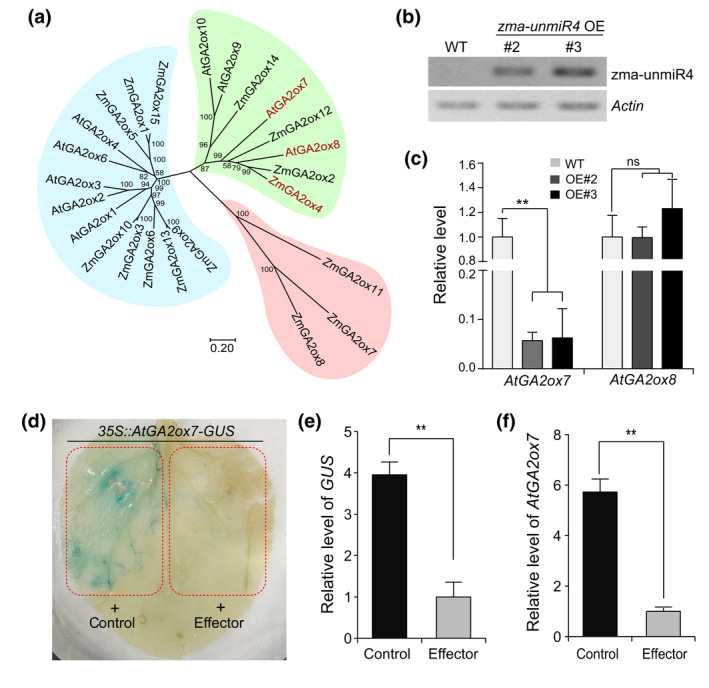
*Arabidopsis AtGA2ox7* is a heterologous target of zma‐unmiR4. (a) Phylogenetic tree of *ZmGA2ox4* homologues in maize and *Arabidopsis*. The sequences of *ZmGA2ox4* homologues from maize and *Arabidopsis* were obtained from the NCBI database (https://blast.ncbi.nlm.nih.gov), and phylogenetic analysis was performed using MEGA 7.0. (b) Measurement of zma‐unmiR4 precursor enrichment in two independent transgenic homozygous lines (*zma‐unmiR4* OE) by RT‐PCR. (c) Expression levels of *AtGA2ox7* or *AtGA2ox8* were quantified in wild‐type (WT) and *zma‐unmiR4* OE plants by reverse transcription‐quantitative PCR. (d) β‐glucuronidase (GUS) staining in tobacco leaves cotransformed with *35S:AtGA2ox7‐GUS* and empty vector or the *35S:pre‐unmiR4* effector as mentioned in panel (d) of Figure 2. (e and f) Quantification of transcript levels of *GUS* (e) and *AtGA2ox7* (f) in the samples described in panel (d). Data are means ± standard deviation of three biological replicates. **p < 0.01 by Student's *t* test; ns, no significant difference.

*Arabidopsis AtGA2ox7* and *AtGA2ox8*, encoding homologous proteins of ZmGA2ox4, were predicted to be the putative heterologous targets of zma‐unmiR4 (Figures 3a and S2). We compared the expression changes of *AtGA2ox7* or *AtGA2ox8* between wild‐type (WT) and zma‐unmiR4‐overexpressing (*zma‐unmiR4* OE) plants (Figure 3b). As shown in Figure 3c, *AtGA2ox7* was significantly down‐regulated while *AtGA2ox8* displayed no obvious changes in both zma‐unmiR4 overexpressors, suggesting that *AtGA2ox7* may be targeted by zma‐unmiR4. To verify this regulation in planta, *AtGA2ox7* was fused with the gene encoding GUS, and this fusion gene was transiently coexpressed with *35S:pre‐unmiR4* in tobacco. GUS activity and transcript levels were dramatically decreased compared with the vector control (Figure 3d,e), and *AtGA2ox7* transcript levels were greatly reduced (Figure 3f). These data demonstrate that zma‐unmiR4 negatively regulates *AtGA2ox7* in *Arabidopsis*.

### 2.3 Overexpression of zma‐unmiR4 confers *Arabidopsis* growth and *F. verticillioides* susceptibility

**FIGURE 4:**
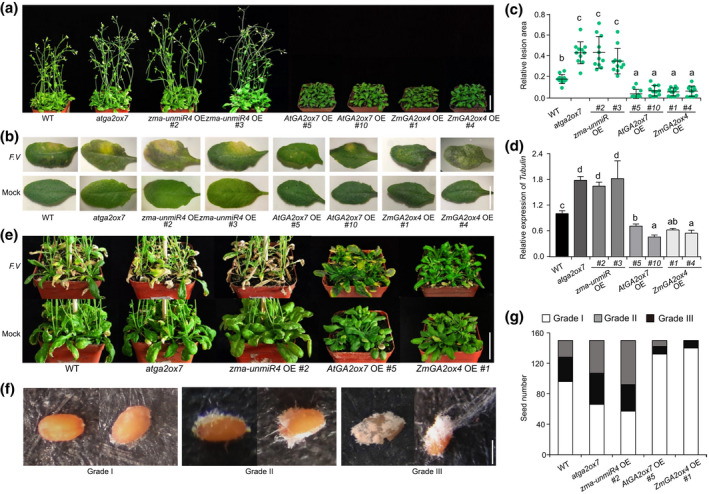
zma‐unmiR4 negatively regulates *Fusarium verticillioides* resistance and positively regulates growth through the target genes *AtGA2ox7* and *ZmGA2ox4*. (a) Growth phenotypes of wild‐type (WT), *atga2ox7* mutant, *zma‐unmiR4* overexpressing (OE), *AtGA2ox7* OE, and *ZmGA2ox4* OE plants. Five‐week‐old *Arabidopsis* seedlings were photographed. Bar = 3 cm. (b) Disease symptoms on representative leaves of different genotypes at 5 days postinoculation. Healthy rosette leaves of 4‐week‐old plants were inoculated with 20 μl *F. verticillioides* spore suspension (*F. V*) or sterile water (Mock). Bar = 1 cm. (c) Investigation of the relative lesion area in the inoculated leaves of indicated genotypes. The relative lesion area (lesion area/total area of each leaf) was measured by ImageJ software. Ten leaves from three biological replicates were analysed for each genotype. (d) Investigation of *F. verticillioides* content in the inoculated leaves of indicated genotypes. *F. verticillioides tubulin* level determined by quantitative PCR was used as an indicator of *F. verticillioides* content. *Arabidopsis Actin 2* was used as internal control. (e) Disease symptoms of different *Arabidopsis* genotypes after *F. verticillioides* spraying. Four‐week‐old plants were sprayed with a *F. verticillioides* spore suspension or sterile water. Bar = 3 cm. (f) Disease grades of *F. verticillioides*‐caused seed rot. Healthy dry seeds were sterilized, immersed in *F. verticillioides* spore suspension for 48 h, and placed on sterile filter paper for 6 days for disease grade investigation. Three disease grades were classified according to the infected area (IA, the area covered by mycelia on one seed/the total area of the same seed): grade I, 0 < IA ≤ 25%; grade II, 25% < IA ≤ 50%; grade III, 50% < IA ≤ 100%. Bar = 250 μm. (g) Disease grades of seed rot among indicated genotypes. Data are from a total of 150 seeds for each genotype. For panels (c) and (d), data are means ± standard deviation from three biological replicates. Letters above the bars indicate significant differences (*p* < 0.05).

To investigate the biological functions of zma‐unmiR4, we developed homozygous transgenic *Arabidopsis* lines overexpressing zma‐unmiR4 (*zma‐unmiR4* OE, Figure 3b), *ZmGA2ox4* (*ZmGA2ox4* OE; Figure S3a*)*, and *AtGA2ox7* (*AtGA2ox7* OE; Figure S3b). Interestingly, we found that *zma‐unmiR4* OE plants displayed increased plant height, early flowering, and large leaf size compared with WT plants, which were similar to the phenotypes of *atga2ox7* mutants (SALK_055721C; Figures 4a and S4). In contrast, ectopic expression of *ZmGA2ox4* or *AtGA2ox7* greatly reduced plant height, delayed flowering time, and shortened the leaf radius, and the *AtGA2ox7* OE leaves appeared dark green with higher chlorophyll content (Figures 4a,b and S4), as reported previously (Porri et al., 2012; Shu et al., 2016). In addition, exogenous application of bioactive GA partially rescued the dwarf phenotype of *ZmGA2ox4* OE and *AtGA2ox7* OE plants (Figure S5), indicating a conserved function of *ZmGA2ox4* and *AtGA2ox7* in GA‐mediated plant growth.

We then determined the *F. verticillioides* resistance of plants with various genotypes by inoculating a fungal spore suspension. Five days postinoculation, young leaves of *zma‐unmiR4* OE and *atga2ox7* mutant plants displayed obvious disease symptoms (Figure 4b), and the yellow necrotic lesions were significantly larger than those of WT plants (Figure 4c). In addition, the *F. verticillioides* content was remarkedly increased in *zma‐unmiR4* OE and *atga2ox7* compared with that in WT plants (Figure 4d). By contrast, the leaves of *AtGA2ox7* OE and *ZmGA2ox4* OE plants exhibited slight yellowish necrosis and less *F. verticillioides* enrichment (Figure 4b–d). To check whether the *F. verticillioides* resistance differences could have been caused by different developmental stages among these genotypes, we used batch sowing to ensure that different genotypes at a similar development stage (the time of the first open flower) were selected for *F. verticillioides* inoculation (Figure S6). The same results as in Figure 4b were obtained, suggesting that the differences of *F. verticillioides* resistance were due to the genotypic variation. In addition, the rosette leaves of *zma‐unmiR4* OE and *atga2ox7* plants displayed more severe blight or death phenotypes after spraying with *F. verticillioides* spore suspension; however, the transgenic plants of *ZmGA2ox4* OE or *AtGA2ox7* OE were almost unaffected (Figure 4e).

We further tested whether there existed differences in *F. verticillioides* seed rot among WT, *atga2ox7* mutant, and the transgenic plants indicated above. The seeds from various genotypes were incubated with a *F. verticillioides* spore suspension, and the phenotypes of fungal mycelia growth on the seed surface were recorded after 6 days. Compared to the water treatment control, the growth and invasion areas of fungal mycelia showed remarkable differences among various genotypes after *F. verticillioides* inoculation. The seeds from *zma‐unmiR4* OE or *atga2ox7* mutant plants were more sensitive to *F. verticillioides* but seeds from *ZmGA2ox4* OE or *AtGA2ox7* OE transgenic plants were more resistant (Figure S7). In detail, more than half of the seeds from *zma‐unmiR4* OE and *atga2ox7* mutant plants exhibited disease grades II and III; however, most seeds from *ZmGA2ox4* OE and *AtGA2ox7* OE plants belonged to grade I according to the three grades of disease resistance (Figure 4f,g). These data suggested that zma‐unmiR4 could positively regulate plant growth and negatively regulate *F. verticillioides* resistance by manipulating *AtGA2ox7* or *ZmGA2ox4* expression.

### 2.4 Altered resistance to *F. verticillioides* by zma‐unmiR4 is associated with the production of H_2_O_2_

**FIGURE 5:**
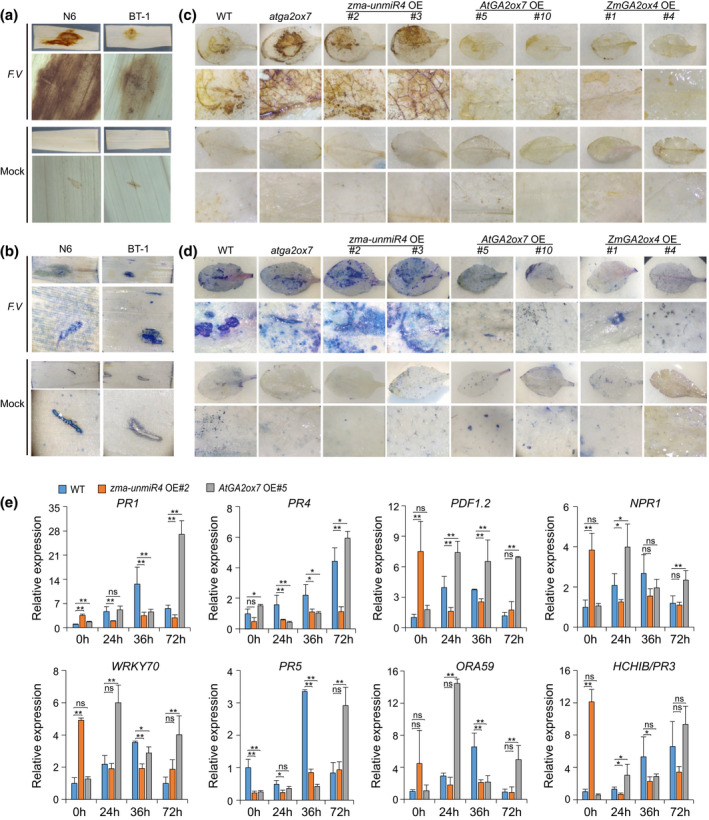
Altered resistance to *Fusarium verticillioides* by zma‐unmiR4 is associated with H_2_O_2_ levels and the expression of defence‐related genes. (a and b) H_2_O_2_ levels and cell death in maize leaves were revealed by 3,3′‐diaminobenzidine (DAB) staining (a) and trypan blue (TB) staining (b), respectively. Healthy second leaves of BT‐1 and N6 maize lines were injected with *F. verticillioides* spore suspension (*F. V*) or sterile water (Mock), and the leaves were sampled for staining at 2 days after inoculation. (c and d) DAB staining (c) and TB staining (d) analysis in *Arabidopsis* leaves of plants with indicated genotypes. WT, wild‐type; OE, overexpression lines. Healthy rosette leaves of 4‐week‐old plants were injected with *F. verticillioides* spore suspension or sterile water and sampled for staining at 4 days after inoculation. The spread of H_2_O_2_ and cell death in the whole leaf are shown in a section of a leaf at 5× magnification. (e) Expression of defence‐related genes in transgenic *Arabidopsis*. Healthy leaves of 4‐week‐old plants were inoculated with a *F. verticillioides* spore suspension and sampled at indicated time points for total RNA extraction. Gene expression levels were quantified by reverse transcription‐quantitative PCR. *Actin 2* was used as internal control. Data are means ± standard deviation of three biological replicates. *p < 0.05, **p < 0.01 by Student's *t* test; ns, no significant difference.

As a necrotrophic fungal pathogen, *F. verticillioides* might ultimately kill and benefit from the infected host cells (Rivas‐San Vicente et al., 2013). Leaves of plants with various genotypes were incubated with 3,3′‐diaminobenzidine (DAB) to detect H_2_O_2_ or stained with trypan blue (TB) to reveal dead cells. We first compared the H_2_O_2_ level and cell death between the leaves of *F. verticillioides*‐susceptible maize line N6 and resistant line BT‐1 after *F. verticillioides* infection. Higher levels of H_2_O_2_ and clusters of dead cells were observed in N6 leaves but lower H_2_O_2_ levels and fewer dead cell clusters were observed in BT‐1 leaves (Figure 5a,b), implying that *F. verticillioides* infection led to an H_2_O_2_ burst and cell death in maize. Consistent with the differences of *F. verticillioides* resistance, we also noted higher H_2_O_2_ levels (Figures 5c and S8) and more cell death (Figure 5d) in *zma‐unmiR4* OE and *atga2ox7* leaves compared with that in WT leaves. On the contrary, cell death was nearly undetectable and a significant reduction of H_2_O_2_ accumulation compared with WT leaves was observed in *ZmGA2ox4* OE and *AtGA2ox7* OE leaves (Figures 5c,d and S8).

### 2.5 Development of *F. verticillioides* resistance by zma‐unmiR4 is correlated with the expression of defence‐related genes

We measured the relative expression levels of a set of defence‐related genes (*pathogenesis‐related 1* [*PR1*], *PR4*, *PR5*, *PDF1.2*, *nonexpressor of PR genes 1* [*NPR1*], *WRKY70*, *ORA59*, and *HCHIB/PR3*) in the transgenic plants indicated above. Notably, the expression levels of these defence‐related genes were significantly increased in response to *F. verticillioides* infection in WT and *AtGA2ox7* OE plants (Figure 5e). In addition, all these genes except for *PR5* were greatly up‐regulated in *AtGA2ox7* OE plants compared to the WT upon *F. verticillioides* infection (Figure 5e). However, for *zma‐unmiR4* OE plants, the expression levels of *PR1*, *PR4*, *PR5*, and *ORA59* displayed no obvious changes after *F. verticillioides* inoculation, and *PDF1.2*, *NPR1*, *WRKY70*, and *HCHIB/PR3* were down‐regulated after *F. verticillioides* inoculation (Figure 5e). These data suggested that zma‐unmiR4‐mediated suppression of *AtGA2ox7* might disturb the induction of defence‐related genes by *F. verticillioides*, thus resulting in resistance variations.

### 2.6 GA accumulation is associated with *F. verticillioides* resistance

**FIGURE 6:**
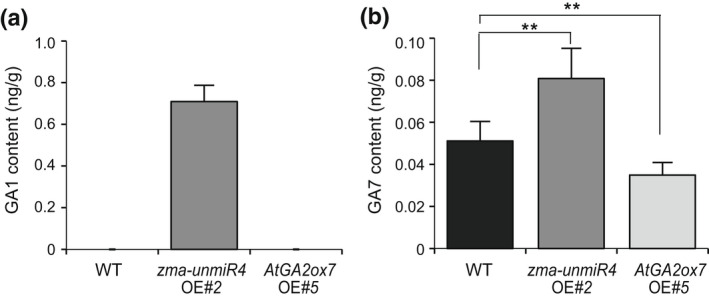
Quantification of endogenous bioactive gibberellins in wild‐type (WT), *zma‐unmiR4* overexpression (OE), and *AtGA2ox7 OE* plants. Shoots of 4‐week‐old *Arabidopsis* plants of various genotypes were sampled for quantifying gibberellins GA1 and GA7 content. Data are means ± standard deviation of three biological replicates. **p < 0.01 by Student's *t* test.

AtGA2ox7, a member of the gibberellin 2‐oxidase family, is a 2‐oxoglutarate‐dependent dioxygenase that regulates the deactivation of bioactive GAs (Li et al., 2019). We analysed the endogenous content of bioactive GA1, GA3, GA4, and GA7 in the rosette leaves of 4‐week‐old plants. The levels of GA3 and GA4 were too low to detect in WT, *zma‐unmiR4* OE, and *AtGA2ox7* OE samples tested, but GA1 accumulated to higher levels in *zma‐unmiR4* OE plants than in WT and *AtGA2ox7* OE plants (Figure 6a). In addition, compared with WT, *zma‐unmiR4* OE transgenic plants accumulated higher levels of GA7 while the contents of GA7 were significantly decreased in *AtGA2ox7* OE (Figure 6b). These results suggested that the zma‐unmiR4–*AtGA2ox7* module mediates plant growth and *F. verticillioides* resistance probably through regulating endogenous bioactive GA accumulation.

**FIGURE 7:**
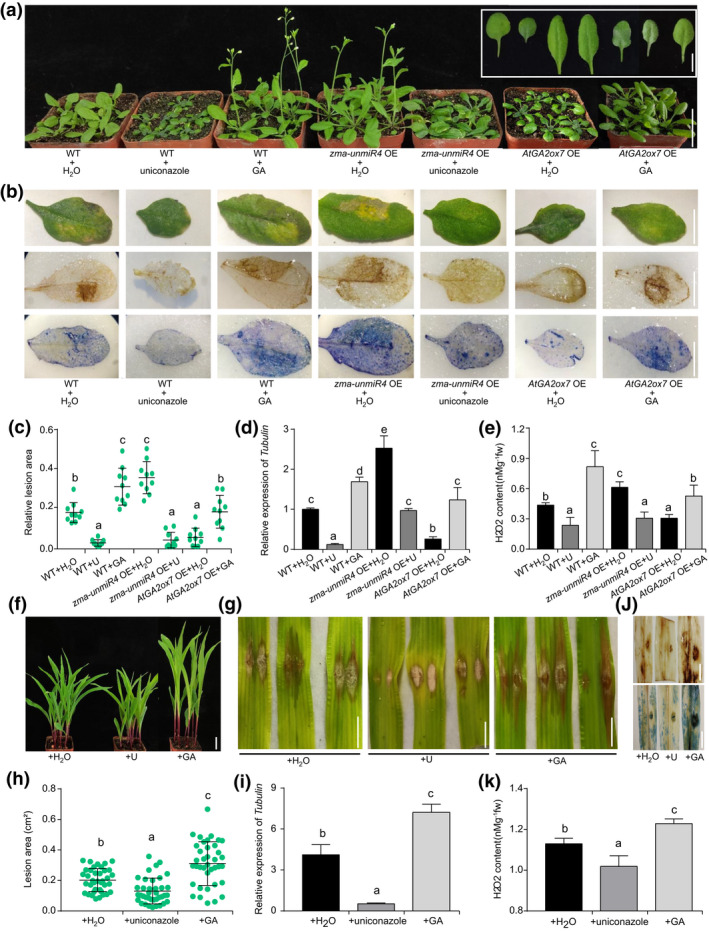
Exogenous application of gibberellin (GA) or GA biosynthesis inhibitor alters plant resistance to *Fusarium verticillioides*. (a) Growth phenotypes of wild‐type (WT), *zma‐unmiR4* overexpression (OE), and *AtGA2ox7 OE* plants. Seventeen‐day‐old *Arabidopsis* plants of indicated genotypes were sprayed with water, GA (50 μM), or uniconazole (U, 20 μM) once a day for 5 days. Bar = 1 cm (insert) or 3 cm (main). (b) Disease symptoms (top), 3,3′‐diaminobenzidine (DAB) staining (middle), and trypan blue (TB) staining (bottom) of representative leaves. Healthy leaves of 17‐day‐old seedlings treated as above were inoculated with a *F. verticillioides* spore suspension and photographed or stained at 4 days after inoculation. Bar = 1 cm. (c) Relative lesion area in the inoculated leaves of indicated treatments. (d) *F. verticillioides* (*Tubulin*) content in the inoculated leaves of indicated treatments. *Arabidopsis Actin 2* was used as the internal control. (e) H_2_O_2_ content in the inoculated leaves of indicated treatments. (f) Growth phenotypes of the susceptible maize line N6 after spraying with water, GA (50 μM), or uniconazole (20 μM). Bar = 3 cm. (g) Disease symptoms of N6 maize leaves. Bar = 1 cm. (h) Relative lesion area in the inoculated maize leaves of indicated treatments. (i) *F. verticillioides* (*Tubulin*) content in the inoculated leaves of indicated treatments. Maize *EF1a* was used as the internal control. (j) DAB staining (top) and TB staining (bottom) of N6 leaves. Bar = 1 cm. (k) H_2_O_2_ content in the inoculated leaves of indicated treatments. For panels (f) to (k), 7‐day‐old seedlings were sprayed with water, GA (50 μM), or uniconazole (20 μM) once a day for 7 days, inoculated with a *F. verticillioides* spore suspension, and photographed, stained, or sampled at 4 days after inoculation. For panels (c) and (h), relative lesion area was measured by ImageJ software, and more than 10 leaves from three biological replicates were analysed for each group. For panels (d) and (i), enrichment of *F. verticillioides Tubulin* as determined by quantitative PCR was used as an indicator of *F. verticillioides* content. Data are means ± standard deviation from three biological replicates. Different letters above the bars indicate significant differences (*p* < 0.05).

To further investigate the effects of GA on plant disease resistance and growth, 17‐day‐old seedlings of WT, *zma‐unmiR4* OE, and *AtGA2ox7* OE transgenes were sprayed with GA or the GA synthesis inhibitor uniconazole. As expected, the growth of WT and *AtGA2ox7* OE seedlings was enhanced by GA treatment, but growth inhibition was observed for both *zma‐unmiR4* OE and WT plants when treated with uniconazole (Figure 7a). We then inoculated the leaves with a *F. verticillioides* spore suspension. Compared to the water control treatment, the leaves of WT and *AtGA2ox7* OE plants treated with GA displayed larger yellow necrotic lesions, significantly increased *F. verticillioides* content, and higher H_2_O_2_ levels as well as cell death (Figure 7b–e). In contrast, both WT and *zma‐unmiR4* OE plants treated with uniconazole exhibited significantly smaller necrotic lesions and less *F. verticillioides* content, and the H_2_O_2_ and cell death levels were much lower than in the water‐treated control (Figure 7b–e).

Moreover, we applied GA or uniconazole on the susceptible maize line N6 to test the changes in resistance to *F. verticillioides*. Compared with the application of water, the susceptibility of N6 seedlings to *F. verticillioides* was greatly promoted by GA, consistent with increased *F. verticillioides* content, necrotic lesions, H_2_O_2_ accumulation, and cell death (Figure 7f–k). In contrast, maize seedlings treated with uniconazole displayed obviously smaller necrotic lesions (Figure 7g,h), significantly less *F. verticillioides* content (Figure 7i), decreased H_2_O_2_ levels (Figure 7j,k), and mild cell death (Figure 7j). Furthermore, similar results were observed in rice seedlings treated with GA and uniconazole (Figure S9). When the rice seedlings were sprayed directly with a *F. verticillioides* spore suspension, the disease symptoms of the seedlings treated with GA were obviously enhanced, while the opposite was observed in seedlings treated with uniconazole (Figure S9). Collectively, these results demonstrate that GA plays a negative role in plants resistance to *F. verticillioides*.

## 3 DISCUSSION

*F. verticillioides* is one of the most common pathogenic fungi and can cause many prevalent diseases in crops, especially in maize, such as seedling blight, root rot, stalk rot, ear rot, and seed rot, leading to poor grain yields and quality, thus posing a great challenge to food and feed safety (Gai et al., 2018; Ju et al., 2017; Mu et al., 2018; Septiani et al., 2019; Zhou et al., 2018). Identification of genes related to *F. verticillioides* resistance and subsequent development of *F. verticillioides*‐resistant crops is considered to be the most economical and environmentally friendly strategy for disease management.

Given that miRNAs provide quantitative regulation of target gene expression rather than switching regulation, the dynamic accumulation of pathogen‐responsive miRNAs can provide fine‐tuning of target gene expression during pathogen infection, thus in turn enhancing the plant's disease resistance (Campo et al., 2013). High‐throughput sequencing of small RNAs is an effective method to discover pathogen‐responsive miRNAs, including conserved and novel miRNAs. Although false‐positive prediction of novel miRNAs cannot be ruled out during sequencing and data processing, the function of these miRNAs in pathogen resistance should be fully considered. For instance, Md‐miRln20 (Zhang et al., 2019), osa‐miR7695 (Campo et al., 2013), and Md‐miRLn11 (Ma et al., 2014) were characterized by small RNA sequencing and their function in disease resistance was experimentally validated. In a previous study, multiple *F. verticillioides*‐responsive miRNAs were identified using small RNA deep sequencing (Zhou et al., 2020), and one of the novel miRNAs, zma‐unmiR4, displayed entirely different expression patterns between susceptible and resistant maize lines after *F. verticillioides* infection (Figure 1a–c). RNA blotting provided evidence for the existence of zma‐unmiR4 in maize (Figure 1d). The significant reduction in zma‐unmiR4 expression in the resistant line BT‐1 upon *F. verticillioides* exposure indicated that it may function as a negative regulator of maize immunity against *F. verticillioides* (Figure 1a–c), manifested by the compromised resistance of transgenic plants ectopically expressing zma‐unmiR4 in *Arabidopsis* (Figure 4). The divergent expression pattern of zma‐unmiR4 between BT‐1 and N6 after *F. verticillioides* infection may be due to the different promoter regions. We sequenced the approximately 900‐bp fragment upstream of zma‐unmiR4, and found that there were 41 single‐nucleotide polymorphisms and a 23‐bp deletion in BT‐1 compared with N6 (Figure S10a), and thus many differences in *cis*‐acting regulatory DNA elements (https://www.dna.affrc.go.jp/PLACE/). We also observed differences of GA‐related growth phenotypes between BT‐1 and N6: BT‐1 had a higher plant height and longer leaves than N6 at the same time after sowing (Figure S10b), which was consistent with the higher expression level of zma‐unmiR4 and the lower expression level of *ZmGA2ox4* in BT‐1 under normal conditions (Figures 1a–c and 2b). Upon *F. verticillioides* infection, zma‐unmiR4 was down‐regulated in BT‐1, probably due to the polymorphisms in its promoter, thus leading to an increase of *ZmGA2ox4* expression, which might be ultimately beneficial for *F. verticillioides* resistance.

According to the different life styles, plant pathogens can be divided into biotrophs (which prefer living cells) and necrotrophs (which prefer dead cells) (Barna et al., 2012). In the case of necrotrophic pathogens such as *Botrytis cinerea*, cell death and tissue necrosis caused by accumulation of reactive oxygen species during pathogen infection were reported to benefit pathogen invasion by offering a growth substrate, thus increasing host susceptibility (Hanif et al., 2018; Tian et al., 2019; Wang et al., 2018). *F. verticillioides* is a necrotrophic fungal pathogen (Rivas‐San Vicente et al., 2013). In line with this, more cell death and higher H_2_O_2_ levels were detected in the leaves of the susceptible maize line N6 compared with the resistant line BT‐1 after *F. verticillioides* infection (Figure 5). Similarly, the susceptibility to *F. verticillioides* infection was also correlated with the cell death and H_2_O_2_ levels in WT, *zma‐unmiR4* OE, *AtGA2ox7* OE, and *ZmGA2ox4* OE plants (Figures 4, 5 and S8). Therefore, cell death and H_2_O_2_ accumulation can be used as indicators of *F. verticillioides* susceptibility in maize cultivars.

GAs are phytohormones that play multiple roles in plant development and stress responses (Rizza & Jones, 2019; Schomburg et al., 2003). Endogenous levels of bioactive GAs are maintained through a balance of biosynthesis and inactivation. AtGA2ox7 is a 2‐oxoglutarate‐dependent dioxygenase that regulates the deactivation of bioactive GAs (Li et al., 2019). Consistently, transgenic plants overexpressing *AtGA2ox7* showed a significant reduction of bioactive GAs compared to the WT, thus exhibiting GA‐deficient phenotypes such as dwarfism, delayed flowering, and small dark green leaves (Porri et al., 2012; Schomburg et al., 2003; Shu et al., 2016) (Figures 4a,b, 6 and S4). By contrast, dysfunction of *AtGA2ox7* resulted in GA‐induced phenotypes, including enhanced growth, large leaf size, and early flowering (Magome et al., 2008; Rieu et al., 2008; Shu et al., 2016), which was consistent with the phenotypes of *zma‐unmiR4* OE plants and higher GA contents (Figures 4a,b, 6 and S4). Therefore, we have reason to believe that the high level of bioactive GAs by zma‐unmiR4‐mediated repression of *AtGA2ox7* is responsible for the phenotypic changes of *zma‐unmiR4* OE plants.

Although the function of bioactive GA in plant growth and development is well known, the role of GA in plant resistance to *F. verticillioides* remains unclear. In fact, GA was first identified from *Gibberella fujikuroi* (*Fusarium moniliforme*), a necrotrophic fungus that causes rice bakanae disease (Yabuta & Sumiki, 1938). Overexpression of the GA‐deactivating enzyme Eui can increase resistance to bacterial blight and rice blast caused by *X. oryzae* and *M. oryzae*, respectively; however, transgenic rice overexpressing *OsGA20ox3* (encoding a GA biosynthesis enzyme) was more susceptible to both diseases (Qin et al., 2013; Yang et al., 2008). Similarly, our current results through genetic and physiological analysis in *Arabidopsis* or maize plants demonstrated that GAs also exhibit a negative effect on the resistance to *F. verticillioides* (Figures 4 and 7). Modification of the expression levels of *AtGA2ox7* or *ZmGA2ox4*, which encode GA‐deactivating enzymes, could change *F. verticillioides* resistance (Figure 4). Despite the enhanced resistance to *F. verticillioides* upon overaccumulation of *AtGA2ox7* in *Arabidopsis*, many adverse effects on development were observed, such as dwarfism and delayed flowering (Figure 4a), which would also be expected to occur in maize. Application of the GA biosynthesis inhibitor uniconazole significantly reduces the lodging rate and enhances yield in maize (Ahmad et al., 2021). Our data also showed the positive effects of uniconazole on *F. verticillioides* resistance and dwarf traits in maize and rice (Figure 7f–k and S9). Therefore, fine‐tuning of the zma‐unmiR4–ZmGA2ox4 regulatory module could theoretically be an alternative way to generate desirable resistance to *F. verticillioides* and to lodging without growth or yield penalty in maize breeding.

**FIGURE 8:**
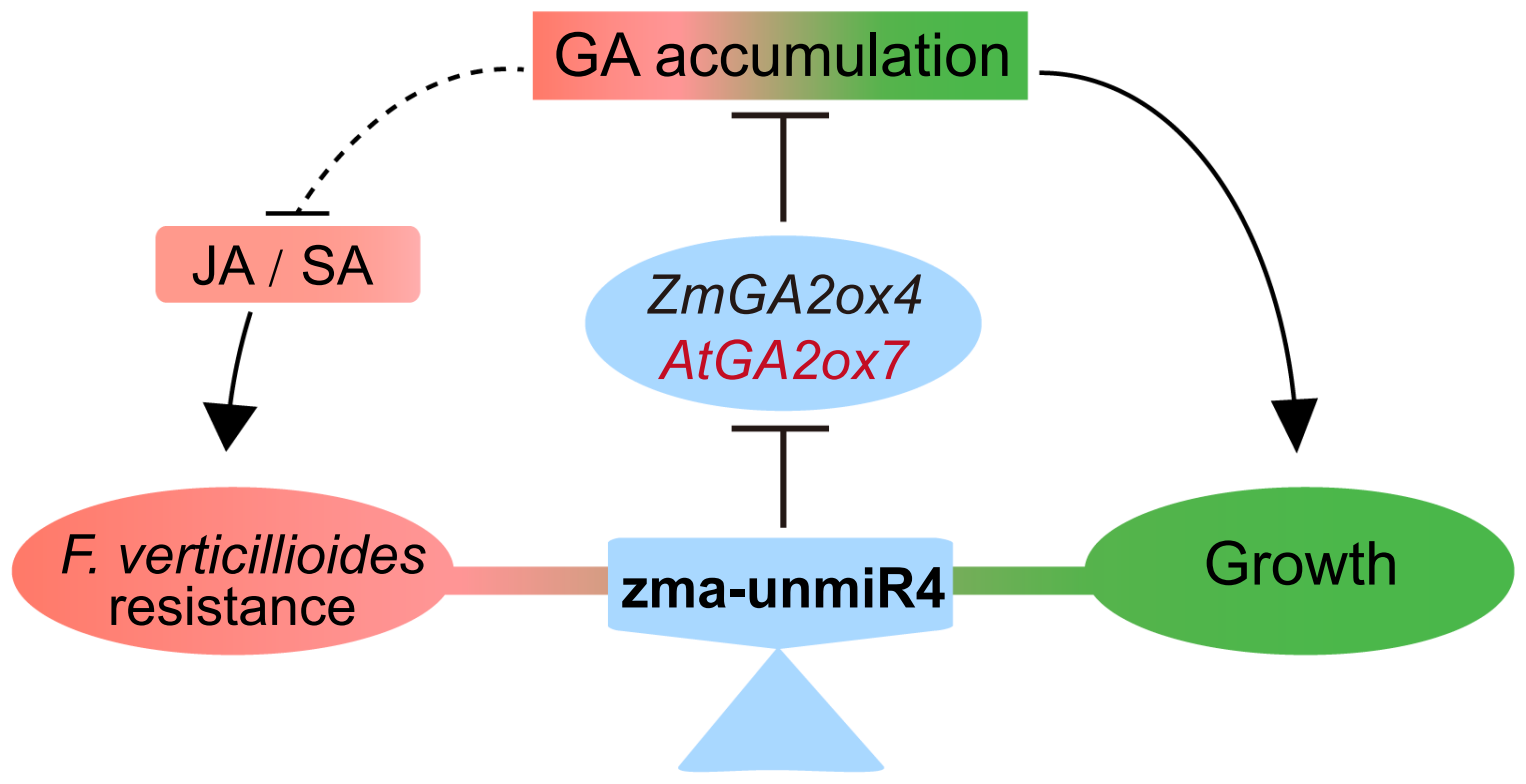
A hypothetical model of zma‐unmiR4 in mediating plant resistance to *Fusarium verticillioides* and growth. zma‐unmiR4 suppresses *ZmGA2ox4* and *AtGA2ox7* expression by stimulating transcript cleavage, leading to bioactive gibberellin (GA) accumulation. On the one hand, increased GA levels perturb jasmonic acid (JA)/salicylic acid (SA)‐mediated defence signalling, thereby resulting in compromised *F. verticillioides* resistance. On the other hand, increased bioactive GA levels confer plant growth and development. Thus, manipulating the zma‐unmiR4–ZmGA2ox4 module may represent an alternative strategy that provides a better balance between *F. verticillioides* disease resistance and growth in maize.

Given the crucial role of GA in plant innate immunity (De Vleesschauwer et al., 2016; Qin et al., 2013; Yang et al., 2008), it is not surprising to find the divergent resistance to *F. verticillioides* between the GA‐deficient (*AtGA2ox7* OE) and GA‐sufficient (*zma‐unmiR4* OE) plants (Figures 4, 5, and 8). On the one hand, GA may give rise to an indirect attenuation of PR genes, thus facilitating pathogen invasion. Indeed, the expression of *PR1*, *PR3*, *PR4*, and *PR5* was significantly induced in response to *F. verticillioides* infection in *AtGA2ox7* OE plants; however, these genes displayed no obvious changes in expression after *F. verticillioides* inoculation in *zma‐unmiR4* OE plants (Figure 5e). GA may hinder disease defence responses by modulating the homeostasis of the archetypal immunity hormones (Verma et al., 2016; Wild & Achard, 2013) such as salicylic acid (SA), jasmonic acid (JA), and ethylene (ET). In rice, overexpression or dysfunction of the GA‐deactivating enzyme Eui results in disturbed homeostasis of SA and JA, thus leading to altered disease susceptibility (Yang et al., 2008). In addition, the SA receptor‐encoding gene *NPR1* is a master regulator of systemic acquired resistance in plants, and overaccumulation of NPR1 leads to enhanced disease resistance to diverse pathogens (Ding et al., 2018). In support of this notion, we found that *NPR1* expression was significantly induced after *F. verticillioides* infection in *AtGA2ox7* OE plants but repressed in *zma‐unmiR4* OE plants (Figure 5e), implying divergent SA signalling dynamics in those transgenic plants upon pathogen attack. Moreover, NPR1 might activate several WRKY transcription factors, such as WRKY70, subsequently leading to massive induction of antimicrobial genes (Saleh et al., 2015). As expected, *F. verticillioides*‐induced expression of *WRKY70* in WT, *zma‐unmiR4* OE, and *AtGA2ox7* OE plants was similar to that of *NPR1* (Figure 5e). Additionally, the divergent expression profiles of *ORA59* and the JA‐ and ET‐responsive plant defensin gene *PDF1.2* (Zarei et al., 2011) further revealed the different dynamics of JA‐ and ET‐associated resistance responses in the transgenic plants upon *F. verticillioides* exposure. Further genome‐wide transcriptome analysis of *zma‐unmiR4* OE and *AtGA2ox7* OE plants should provide much‐needed insights into the interactions between GA and other phytohormone signalling pathways that underpin plant resistance in response to *F. verticillioides* challenge.

In conclusion, we discovered a novel *F. verticillioides‐*responsive miRNA, zma‐unmiR4, in maize kernels and revealed that *ZmGA2ox4* and its homologue *AtGA2ox7* are the targets of zma‐unmiR4. We showed that zma‐unmiR4‐mediated suppression of *AtGA2ox7* disturbed the accumulation of bioactive GA, and zma‐unmiR4–*ZmGA2ox4/AtGA2ox7*‐mediated bioactive GA dynamics act as a crucial regulator in *F. verticillioides* resistance and plant growth (Figure 8). However, as a master regulator of plant growth and development, GA confers susceptibility to multiple plant diseases (Qin et al., 2013; Yang et al., 2008; Zhang et al., 2020). Our study provides a new strategy for a better balance between *F. verticillioides* disease resistance and growth in maize breeding by engineering the zma‐unmiR4–*ZmGA2ox4* module.

## 4 EXPERIMENTAL PROCEDURES

### 4.1 Plant materials and growth conditions

The *F. verticillioides*‐susceptible maize inbred line N6 is a Tangsipingtou line, whereas the *F. verticillioides*‐resistant maize line BT‐1 is improved by tropical Asia material (Wang et al., 2016). Seedlings were grown at 25 ± 2°C with a 14/10‐h light/dark photoperiod. *Arabidopsis* (Col‐0) seeds were first sterilized with 75% ethanol and then soaked in 3% sodium hypochlorite. Surface‐sterilized seeds were stratified in the dark at 4°C for 2 days and sown on Murashige–Skoog dishes (pH 5.7) for 7 days. Then seedlings were transferred to sterilized nutritional soil at 22°C with a 16/8‐h light/dark photoperiod. The healthy seeds of *japonica* rice KY131 were soaked in 3% sodium hypochlorite, germinated at 37°C for 3 days, sown into 96‐well plates, and water‐cultured at 28 ± 2°C with a 14/10‐h light/dark photoperiod.

### 4.2 Vector construction and generation of transgenic *Arabidopsis* plants

For *zma‐unmiR4* overexpression, the 398‐bp hairpin region of *zma‐unmiR4* was amplified from genomic DNA of N6 and ligated into pJim19(Bar) driven by a CaMV 35S promoter. For *ZmGA2ox4* and *AtGA2ox7* overexpression, the coding sequences of *ZmGA2ox4* and *AtGA2ox7* were ligated into pCANBIA1302 (HYG), driven by a 35S promoter. After confirmation by sequencing, the vectors were introduced into *Agrobacterium tumefaciens* GV3101 and transferred into Col‐0 by floral dip. For *35S::ZmGA2ox4‐GUS*, *35S::ZmGA2ox4M‐GUS*, and *35S::AtGA2ox7‐GUS* vectors, the coding sequences of *ZmGA2ox4*, mutant *ZmGA2ox4*, and *AtGA2ox7* without a stop codon were ligated into the binary vector pCAMBIA1391 (HYG) driven by a 35S promoter. For the *35S::ZmGA2ox4‐YFP* vector, the coding sequences of *ZmGA2ox4* without a stop codon were ligated into pGRDR driven by a 35S promoter. For the transient expression of *zma‐unmiR4* in maize protoplasts, the 398‐bp hairpin region of *zma‐unmiR4* was amplified from genomic DNA of N6 and ligated into pPROTO driven by a 35S promoter. The primers used for vector construction are listed in Table S1.

### 4.3 Transfection of maize protoplasts

Maize protoplasts were isolated as previously described (Li et al., 2021). *35S::ZmGA2ox4‐YFP* was cotransfected into the protoplasts with *35S::pre‐zma‐unmiR4* or empty vector. After incubation in the dark for 16 h, YFP and mCherry signals were observed using a laser scanning confocal microscope (A1HD25; Nikon). The relative fluorescence intensity (YFP/mCherry) was calculated by ImageJ software.

### 4.4 *F. verticillioides* inoculation and phenotype investigation

The *F. verticillioides* strain was isolated from naturally infected maize kernels in Zhengzhou. A single spore of *F. verticillioides* was isolated and propagated on sterilized maize kernels at 28°C for 7 days. The spores were then collected and diluted to the indicated concentration using sterile distilled water with 0.2 μl/ml Tween 80. The ear inoculation was performed as previously described (Wu et al., 2020; Zhou et al., 2020). The middle of the ears was injected with 2 ml *F. verticillioides* spore suspension (5 × 10^6^ spores/ml) on the 15th day after pollination.

For *Arabidopsis* leaf inoculation, the healthy rosette leaves at indicated times were inoculated with 20 μl *F. verticillioides* spore suspension (1 × 10^7^ spores/ml). After 4–6 days of culture at 22°C, leaves were photographed or sampled for histological staining, *F. verticillioides* quantification, and H_2_O_2_ content determination. For spore suspension spraying, 5‐week‐old plants were sprayed with a *F. verticillioides* spore suspension (2 × 10^7^ spores/ml) or sterile water once a day for 10 days. For seed inoculation, sterilized seeds were soaked in a *F. verticillioides* spore suspension (1 × 10^7^ spores/ml) at 28°C darkness for 48 h. Then the seeds were evenly placed on wet filter paper in Petri dishes at 28°C in darkness for 6 days.

For maize leaf inoculation, healthy leaves were lacerated with a needle and injected with 10 μl of a *F. verticillioides* spore suspension (1 × 10^7^ spores/ml). After incubation at 25°C for 2–5 days, leaves were photographed or sampled for histological staining, *F. verticillioides* quantification, and H_2_O_2_ content determination.

For rice leaf inoculation, healthy leaves were scratched with a needle, immersed in 3 ml of a *F. verticillioides* spore suspension (2 × 10^7^ spores/ml), and incubated at 25°C for 5 days. For seedling inoculation, healthy seedlings were sprayed with a *F. verticillioides* spore suspension (2 × 10^7^ spores/ml) once a day for 6 days.

### 4.5 RNA analyses

About 1 μg RNA was treated with DNase I (Promega) and reverse transcribed using the Transcriptor First Strand cDNA Synthesis Kit (TOYOBO). The RT‐qPCR assay was performed using SYBR Green I Master reagent and a STEP ONE PLUS system (ThermoFisher). The expression levels of target genes were normalized to the internal control genes using the 2^−ΔΔ*C*t^ method. RNA gel blot analyses of miRNA were performed as described previously (Zhang & Li, 2013). Primer and probe sequences are listed in Table S1.

### 4.6 GUS, DAB, and TB staining and H_2_O_2_ quantification

The transient expression assay for GUS analysis was performed as previously described (Li et al., 2021). For DAB staining, leaves were immersed in 0.1% DAB solution, infiltrated under vacuum conditions for 15 min, and then incubated at room temperature for 12 h in the dark. Chlorophyll was removed by immersion in 95% ethanol. H_2_O_2_ quantification was performed as previously described (Garg et al., 2012). TB staining was performed by submerging the leaves in TB solution (10 ml lactic acid, 10 ml phenol, 10 ml glycerol, 10 ml sterile water, and 10 mg trypan blue) for 30–60 min. Chlorophyll was removed by immersion in 95% ethanol.

### 4.7 Hormone treatments

Seven‐day‐old maize seedlings were sprayed with 20 μM uniconazole, 50 μM GA3, or water once a day for 7 days and photographed, and then leaves were inoculated. Fourteen‐day‐old rice seedlings were sprayed with 20 μM uniconazole, 50 μM GA3, or water once a day for 4 days and then sprayed with a *F. verticillioides* spore suspension. Seventeen‐day‐old *Arabidopsis* seedlings were sprayed with 20 μM uniconazole, 50 μM GA3, or water once a day for 5 days and photographed, and then leaves were inoculated as described above.

### 4.8 Determination of chlorophyll concentration

The measurement of chlorophyll concentration was performed as previously described (Arnon, 1949). Leaves from 5‐week‐old plants were sampled. The absorbance was measured at 663 nm, 645 nm, and 652 nm using an ELISA instrument.

### 4.9 Gibberellin measurement

Healthy rosette leaves of 4‐week‐old plants were harvested, immediately frozen in liquid nitrogen, and ground into powder. Next, 50 mg of plant sample was dissolved in 500 μl HPLC‐grade acetonitrile/water (90:10, vol/vol). As internal standards for the quantification, 10 μl internal standard solution (100 ng/ml) was added into the extract. GA contents were calculated by MetWare (http://www.metware.cn/) based on the AB Sciex QTRAP 6500 liquid chromatography–tandem mass spectrometry platform. Three biological replicates were performed.

## AUTHOR CONTRIBUTIONS

H.Z., Y.X., and J.W. designed the research. R.W., Y.X., Y.C., P.M., J.C., T.L., and Z.Z. performed the experiments. H.Z., Y.X., and T.L. analysed the data. Y.X. and H.Z. prepared the figures and wrote the article. All authors read and approved this manuscript.

## CONFLICT OF INTEREST

The authors declare no conflicts of interest.

## Supporting information


**Figure S1** Tissue‐specific expression of zma‐unmiR4. YR, YS, and YL represent roots, stem, and leaves from 8‐day‐old B73 seedlings, respectively, and stem and leaf were taken from the plants at the flowering stage. Total RNA was extracted from indicated tissues, treated with DNase I, and reverse‐transcribed into cDNA for PCR amplification. *EF1α* was used as a control

**Figure S2** The predicted target sequences of zma‐unmiR4 in *AtGA2ox7* and *AtGA2ox8*. The sequences of *AtGA2ox7* or *AtGA2ox8* transcripts and zma‐unmiR4 were aligned online at the website (http://rna.informatik.uni‐freiburg.de)

**Figure S3** Molecular identification of *ZmGA2ox4 OE* and *AtGA2ox7 OE* transgenic lines. Leaves of 4‐week‐old *ZmGA2ox4 OE* and *AtGA2ox7 OE* transgenic plants were sampled for total RNA extraction, and reverse transcription‐quantitative PCR assays were performed to measure the transcript levels of *ZmGA2ox4* (a) and *AtGA2ox7* (b) genes. *Actin 2* was used as an internal control. Data are means ± standard deviation of three biological replicates. **p < 0.01 by Student’s *t* test

**Figure S4** Quantitative comparison of flowering time, plant height, leaf size, and chlorophyll contents among wild‐type (WT), *atga2ox7* mutant, and transgenic plants. The timing of the first opened flower (a), rosette leaf number at time of the first open flower (b), plant height (c), leaf size (d), leaf area (e), and chlorophyll concentration (f) of WT, *atga2ox7* mutant, *zma‐unmiR4 OE*, *AtGA2ox7 OE*, and *ZmGA2ox4 OE* plants. Five‐week‐old seedlings grown in soil were used for analysis. Data are means ± standard deviation. In panels (a) and (b), *n* = 24 plants; in panel (c), *n* = 20 plants; in panel (e), the largest rosette leaf was used for measuring leaf area by ImageJ software, *n* = 18 plants; in panel (f), *n* = 3 biological replicates. ns, no significant difference; *p < 0.05, **p < 0.01 by Student’s *t* test

**Figure S5** Exogenous gibberellin (GA) partially rescued the dwarf phenotype by *AtGA2ox7* or *ZmGA2ox4* overaccumulation in *Arabidopsis*. Four‐week‐old *AtGA2ox7 OE* and *ZmGA2ox4 OE* transgenic plants grown in soil were sprayed with GA (50 μM) once a day for 10 days and then photographed. Bar = 7 cm

**Figure S6** The disease phenotypes of wild‐type (WT), *atga2ox7* mutant, *zma‐unmiR4 OE*, *AtGA2ox7 OE*, and *ZmGA2ox4 OE* plants at a similar development stage. (a) Growth phenotypes of WT, *atga2ox7* mutant, *zma‐unmiR4 OE*, *AtGA2ox7 OE*, and *ZmGA2ox4 OE* plants at time of the first open flower. Bar = 3 cm. (b) Disease symptoms on representative leaves of plants with indicated genotypes at 5 days postinoculation. Healthy rosette leaves of 33‐day‐old WT, 30‐day‐old *atga2ox7* and *zma‐unmiR4 OE*, and 40‐day‐old *AtGA2ox7 OE* and *ZmGA2ox4 OE* were inoculated with 20 μl *Fusarium verticillioides* spore suspension (*F. V*) or sterile water (Mock). Bar = 1 cm. (c) Relative lesion area in the inoculated leaves of indicated genotypes. The relative lesion area (lesion area/total area of each leaf) was measured by ImageJ software. More than 10 leaves were analysed for each genotype. Letters above the bars indicate significant differences (*p* < 0.05 by Student’s *t* test)

**Figure S7** The seed rot symptoms of wild‐type (WT), *atga2ox7* mutant, *zma‐unmiR4 OE*, *AtGA2ox7 OE*, and *ZmGA2ox4 OE* plants. Healthy dry seeds were sterilized, immersed in *Fusarium verticillioides* spore suspension (*F. V*) or sterile water (Mock) for 48 h, and placed in sterile filter paper for 6 days

**Figure S8** Investigation of H_2_O_2_ content in the inoculated leaves of indicated genotypes. Healthy rosette leaves of 4‐week‐old plants were inoculated with 20 μl *Fusarium verticillioides* spore suspension and then sampled at 5 days postinoculation for determination of H_2_O_2_ content. Data are means ± standard deviation from three biological replicates. Letters above the bars indicate significant differences (*p* < 0.05 by Student’s *t* test)

**Figure S9** Exogenous application of gibberellin (GA) alters rice resistance to *Fusarium verticillioides*. (a) Growth phenotype of KY131 rice seedlings. Fourteen‐day‐old seedlings were sprayed with H_2_O, GA (50 μM), or uniconazole (20 μM) once a day for 4 days and then photographed. Bar = 5 cm. (b, c) Disease symptoms of KY131 leaves upon *F. verticillioides* exposure. Bar = 1 cm. For panel (b), 14‐day‐old seedlings were treated as in panel (a) and then sprayed with *F. verticillioides* spore suspension (*F. V*) and sterile water (Mock) once a day for 6 days and photographed. For panel (c), 14‐day‐old seedlings were treated as in panel (a), and then the leaves were immersed in *F. V* spore suspension for 5 days and photographed

**Figure S10** Seedling growth difference and the promoter sequence divergence of zma‐unmiR4 between BT‐1 and N6. (a) Promoter sequence differences of zma‐unmiR4 between BT‐1 and N6 genotypes. Red letters indicate different bases. The red box indicates the zma‐unmiR4 sequence. (b) Phenotype difference between BT‐1 and N6 seedlings at 7 days after sowing (DAS) and 11 DAS. Bar = 7 cm

**Table S1** Primer sequences used in this study

## Data Availability

The data that support the findings of this study are available from the corresponding author upon reasonable request.
