# Supplementary material for: A novel maize microRNA negatively regulates resistance to Fusarium verticillioides
Source: Mol Plant Pathol. 2022 Jun 14;23(10):1446–60. doi: 10.1111/mpp.13240 (PMC9452762; doi:10.1111/mpp.13240)
Supplement: Supplementary file 1 — Figure S1 Tissue‐specific expression of zma‐unmiR4. YR, YS, and YL represent roots, stem, and leaves from 8‐day‐old B73 seedlings, respectively, and stem and leaf were taken from the plants at the flowering stage. Total RNA was extracted from indicated tissues, treated with DNase I, and reverse‐transcribed into cDNA for PCR amplification. EF1α was used as a control [file MPP-23-1446-s002.docx]

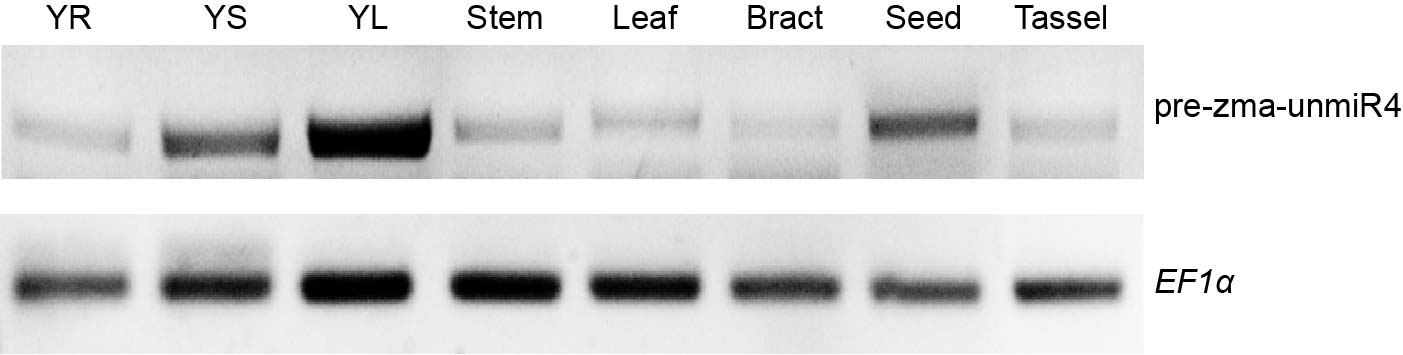


**Figure S1. Tissue-specific expression of zma-unmiR4.**

YR, YS and YL represented roots, stem and leaves from 8-day-old B73 seedlings, respectively, and stem and leaf were taken from the plants at the flowering stage. Total RNA was extracted from indicated tissues and treated with DNase I, and reverse-transcribed into cDNA for PCR amplification. *EF1α* was used as a control.
