# Supplementary material for: A novel maize microRNA negatively regulates resistance to Fusarium verticillioides
Source: Mol Plant Pathol. 2022 Jun 14;23(10):1446–60. doi: 10.1111/mpp.13240 (PMC9452762; doi:10.1111/mpp.13240)
Supplement: Supplementary file 2 — Figure S2 The predicted target sequences of zma‐unmiR4 in AtGA2ox7 and AtGA2ox8. The sequences of AtGA2ox7 or AtGA2ox8 transcripts and zma‐unmiR4 were aligned online at the website (http://rna.informatik.uni‐freiburg.de) [file MPP-23-1446-s010.docx]

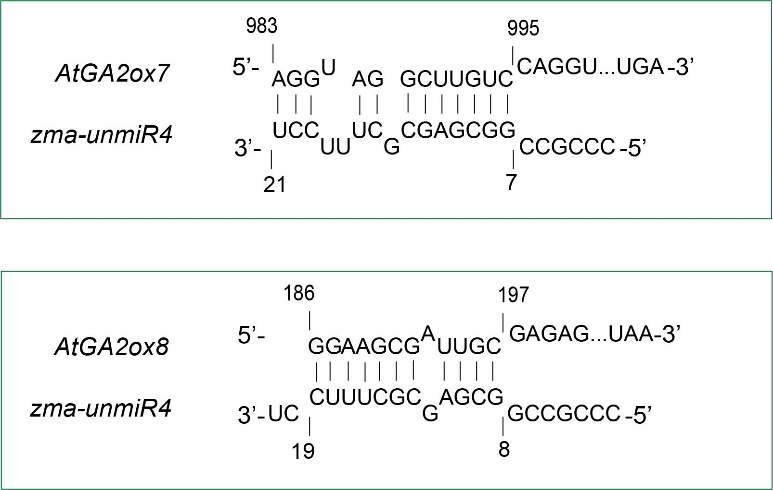


**Figure S2. The predicted target sequences of zma-unmiR4 in** ***AtGA2ox7* and *AtGA2ox8*.**

The sequences of *AtGA2ox7* or *AtGA2ox8* transcripts and zma-unmiR4 were aligned online at the website (http://rna.informatik.uni-freiburg.de).
