# Supplementary material for: A novel maize microRNA negatively regulates resistance to Fusarium verticillioides
Source: Mol Plant Pathol. 2022 Jun 14;23(10):1446–60. doi: 10.1111/mpp.13240 (PMC9452762; doi:10.1111/mpp.13240)
Supplement: Supplementary file 3 — Figure S3 Molecular identification of ZmGA2ox4 OE and AtGA2ox7 OE transgenic lines. Leaves of 4‐week‐old ZmGA2ox4 OE and AtGA2ox7 OE transgenic plants were sampled for total RNA extraction, and reverse transcription‐quantitative PCR assays were performed to measure the transcript levels of ZmGA2ox4 (a) and AtGA2ox7 (b) genes. Actin 2 was used as an internal control. Data are means ± standard deviation of three biological replicates. **p < 0.01 by Student’s t test [file MPP-23-1446-s007.docx]

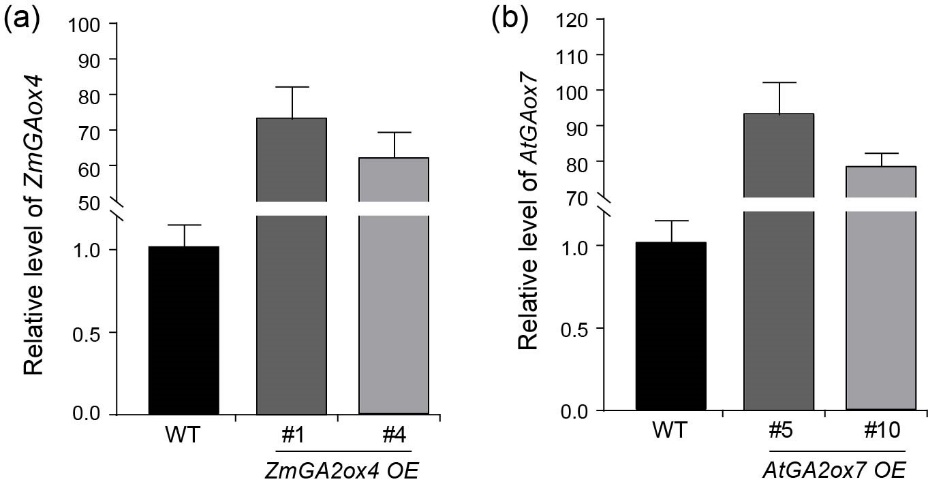


**Figure S3. Molecular identification of** ***ZmGA2ox4 OE* and *AtGA2ox7 OE* transgenic lines.**

Leaves of 4-week-old *ZmGA2ox4 OE* and *AtGA2ox7 OE* transgenic plants were sampled for total RNA extraction, and reverse transcription‐quantitative PCR assays were performed to measure the transcript levels of *ZmGA2ox4* (a) and *AtGA2ox7* (b) genes. *Actin2* was used as an internal control. Data are means ± SD of three biological replicates. ** *P* < 0.01 by Student’s *t* test.
