# Supplementary material for: A novel maize microRNA negatively regulates resistance to Fusarium verticillioides
Source: Mol Plant Pathol. 2022 Jun 14;23(10):1446–60. doi: 10.1111/mpp.13240 (PMC9452762; doi:10.1111/mpp.13240)
Supplement: Supplementary file 4 — Figure S4 Quantitative comparison of flowering time, plant height, leaf size, and chlorophyll contents among wild‐type (WT), atga2ox7 mutant, and transgenic plants. The timing of the first opened flower (a), rosette leaf number at time of the first open flower (b), plant height (c), leaf size (d), leaf area (e), and chlorophyll concentration (f) of WT, atga2ox7 mutant, zma‐unmiR4 OE, AtGA2ox7 OE, and ZmGA2ox4 OE plants. Five‐week‐old seedlings grown in soil were used for analysis. Data are means ± standard deviation. In panels (a) and (b), n = 24 plants; in panel (c), n = 20 plants; in panel (e), the largest rosette leaf was used for measuring leaf area by ImageJ software, n = 18 plants; in panel (f), n = 3 biological replicates. ns, no significant difference; *p < 0.05, **p < 0.01 by Student’s t test [file MPP-23-1446-s006.docx]

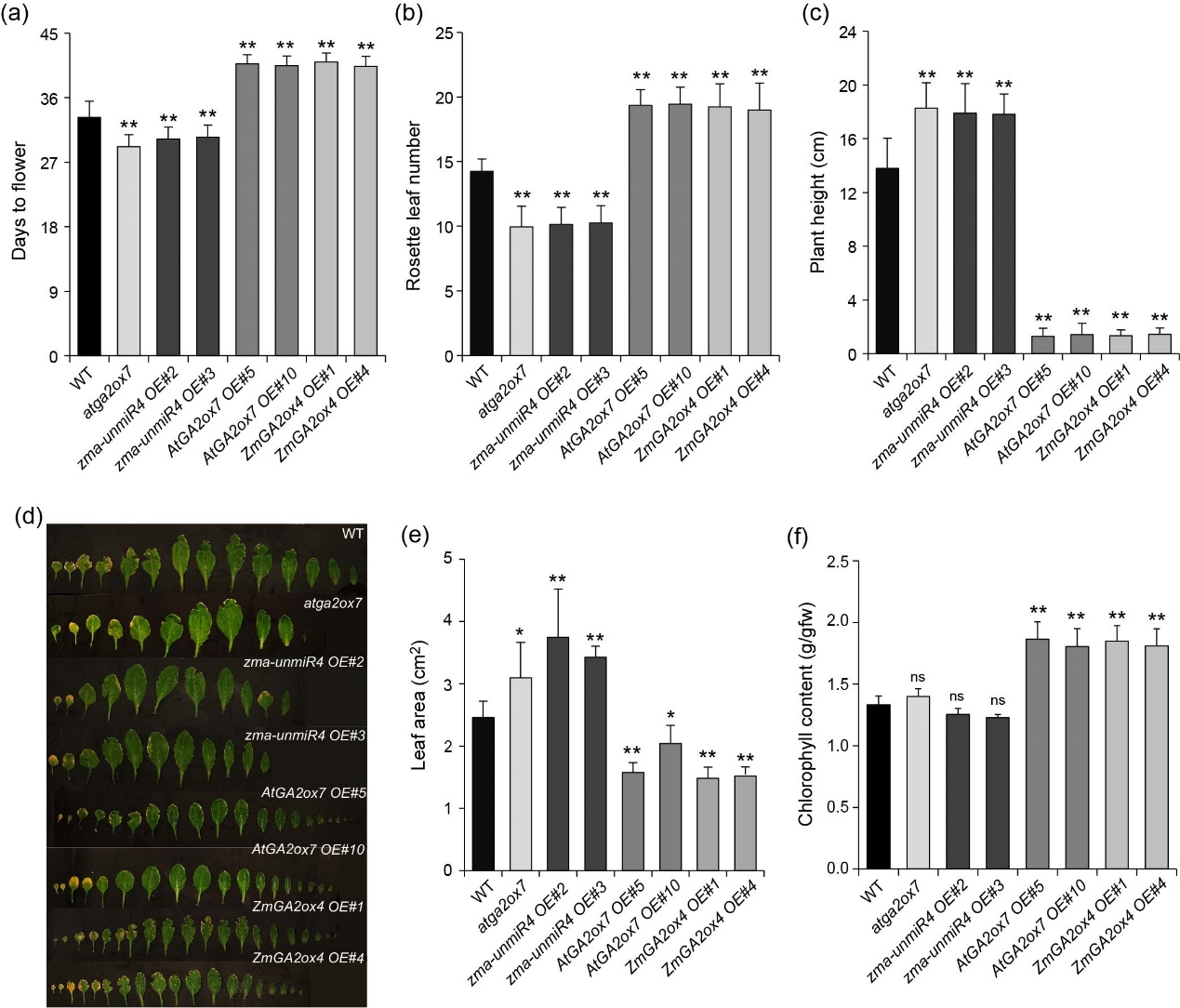


**Figure S4. Quantitative comparison of flowering time, plant height, leaf size and** **chlorophyll contents among wild‐type (WT), *atga2ox7* mutant, and transgenic plants.**

The timing of the first opened flower (a), rosette leaf number at time of the first open flower (b), plant height (c), leaf size (d), leaf area (e), and chlorophyll concentration (f) of WT, *atga2ox7* mutant, *zma-unmiR4 OE*, *AtGA2ox7 OE*, and *ZmGA2ox4 OE* plants. Five-week-old seedlings grown in soil were used for analysis. Data are means ± SD. Panel a and b, n = 24 plants; Panel c, n = 20 plants; Panel e, the largest rosette leaf was used for measuring leaf area by Image J software, n = 18 plants. Panel f, n = three biological replicates. ns, no significant difference; * *P* < 0.05, ** *P* < 0.01 by Student’s *t* test.
