# Supplementary material for: A novel maize microRNA negatively regulates resistance to Fusarium verticillioides
Source: Mol Plant Pathol. 2022 Jun 14;23(10):1446–60. doi: 10.1111/mpp.13240 (PMC9452762; doi:10.1111/mpp.13240)
Supplement: Supplementary file 5 — Figure S5 Exogenous gibberellin (GA) partially rescued the dwarf phenotype by AtGA2ox7 or ZmGA2ox4 overaccumulation in Arabidopsis. Four‐week‐old AtGA2ox7 OE and ZmGA2ox4 OE transgenic plants grown in soil were sprayed with GA (50 μM) once a day for 10 days and then photographed. Bar = 7 cm [file MPP-23-1446-s009.docx]

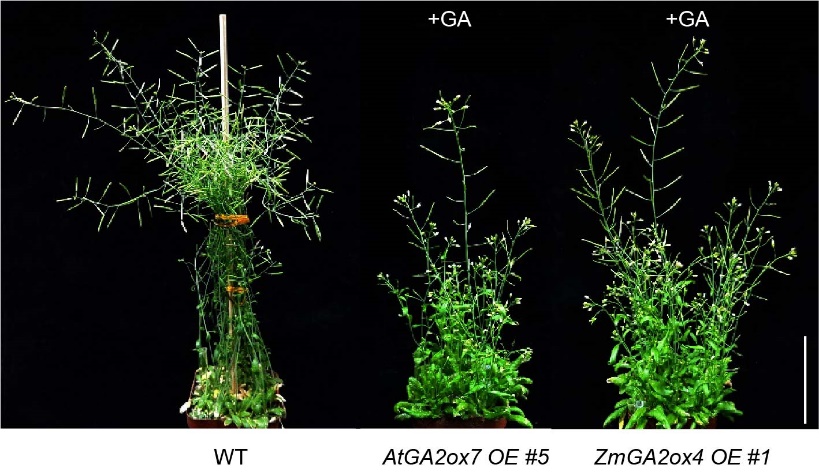


**Figure S5. Exogenous gibberellin (GA) partially rescued the dwarf phenotype by *AtGA2ox7* or *ZmGA2ox4* overaccumulation in *Arabidopsis*.**

Four-week-old *AtGA2ox7* OE and *ZmGA2ox4* OE transgenic plants grown in soil were sprayed 10 days, and then photographed. Bar = 7 cm
