# Supplementary material for: A novel maize microRNA negatively regulates resistance to Fusarium verticillioides
Source: Mol Plant Pathol. 2022 Jun 14;23(10):1446–60. doi: 10.1111/mpp.13240 (PMC9452762; doi:10.1111/mpp.13240)
Supplement: Supplementary file 6 — Figure S6 The disease phenotypes of wild‐type (WT), atga2ox7 mutant, zma‐unmiR4 OE, AtGA2ox7 OE, and ZmGA2ox4 OE plants at a similar development stage. (a) Growth phenotypes of WT, atga2ox7 mutant, zma‐unmiR4 OE, AtGA2ox7 OE, and ZmGA2ox4 OE plants at time of the first open flower. Bar = 3 cm. (b) Disease symptoms on representative leaves of plants with indicated genotypes at 5 days postinoculation. Healthy rosette leaves of 33‐day‐old WT, 30‐day‐old atga2ox7 and zma‐unmiR4 OE, and 40‐day‐old AtGA2ox7 OE and ZmGA2ox4 OE were inoculated with 20 μl Fusarium verticillioides spore suspension (F. V) or sterile water (Mock). Bar = 1 cm. (c) Relative lesion area in the inoculated leaves of indicated genotypes. The relative lesion area (lesion area/total area of each leaf) was measured by ImageJ software. More than 10 leaves were analysed for each genotype. Letters above the bars indicate significant differences (p < 0.05 by Student’s t test) [file MPP-23-1446-s011.docx]

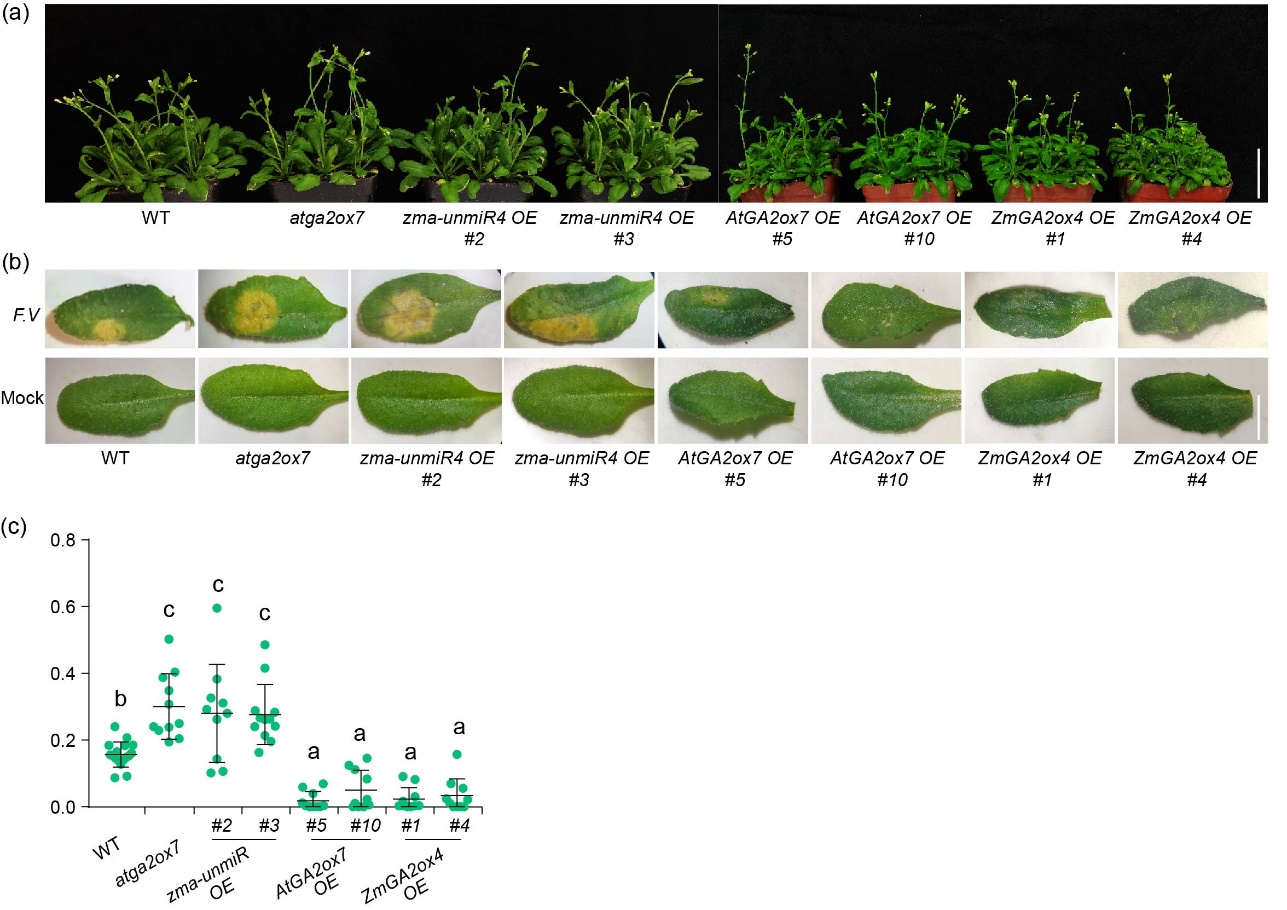


**Figure S6. The disease phenotypes of wild‐type (WT), *atga2ox7* mutant, *zma-unmiR4* OE, *AtGA2ox7* OE, and *ZmGA2ox4* OE plants at the similar development stage.**

**(a)** Growth phenotypes of WT, *atga2ox7* mutant, *zma-unmiR4* OE, *AtGA2ox7* OE, and *ZmGA2ox4* OE plants at time of the first open flower. Bar = 3 cm. **(b)** The disease symptoms on the representative leaves of indicated genotypic plants at 5 days post inoculation. Healthy rosette leaves of 33-day-old WT, 30-day-old *atga2ox7* and *zma-unmiR4* OE, 40-day-old *AtGA2ox7* OE and *ZmGA2ox4* OE, were inoculated with 20 µl *Fusarium verticillioides* spore suspension (*F. V*) or sterile water (Mock). Bar = 1 cm. **(c)** Investigation of the relative lesion area in the inoculated leaves of indicated genotypes. The relative lesion area (lesion area / total area each leaf) was measured by ImageJ software. More than ten leaves were analyzed for each genotype. Letters above the bars indicate significant differences (*P* < 0.05 by Student’s *t* test).
