# Supplementary material for: A novel maize microRNA negatively regulates resistance to Fusarium verticillioides
Source: Mol Plant Pathol. 2022 Jun 14;23(10):1446–60. doi: 10.1111/mpp.13240 (PMC9452762; doi:10.1111/mpp.13240)
Supplement: Supplementary file 7 — Figure S7 The seed rot symptoms of wild‐type (WT), atga2ox7 mutant, zma‐unmiR4 OE, AtGA2ox7 OE, and ZmGA2ox4 OE plants. Healthy dry seeds were sterilized, immersed in Fusarium verticillioides spore suspension (F. V) or sterile water (Mock) for 48 h, and placed in sterile filter paper for 6 days [file MPP-23-1446-s008.docx]

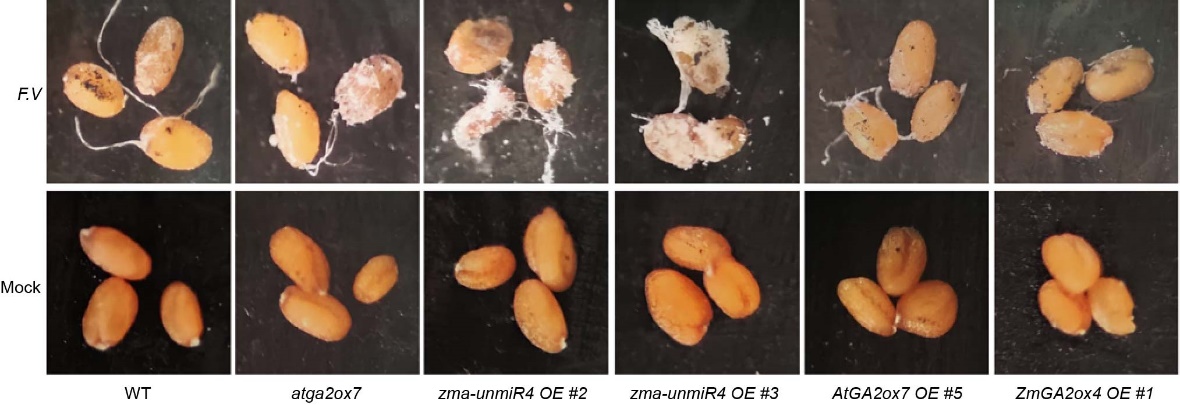


**Figure S7. The seed rot symptoms of wild-type (WT), *atga2ox7* mutant, *zma-unmiR4* OE, *AtGA2ox7* OE, and *ZmGA2ox4* OE plants.**

Healthy dry seeds were sterilized, then immersed in *Fusarium verticillioides* spore suspension (*F. V*) or sterile water (Mock) for 48 h and placed in sterile filter paper for 6 days.
