# Supplementary material for: A novel maize microRNA negatively regulates resistance to Fusarium verticillioides
Source: Mol Plant Pathol. 2022 Jun 14;23(10):1446–60. doi: 10.1111/mpp.13240 (PMC9452762; doi:10.1111/mpp.13240)
Supplement: Supplementary file 8 — Figure S8 Investigation of H2O2 content in the inoculated leaves of indicated genotypes. Healthy rosette leaves of 4‐week‐old plants were inoculated with 20 μl Fusarium verticillioides spore suspension and then sampled at 5 days postinoculation for determination of H2O2 content. Data are means ± standard deviation from three biological replicates. Letters above the bars indicate significant differences (p < 0.05 by Student’s t test) [file MPP-23-1446-s005.docx]

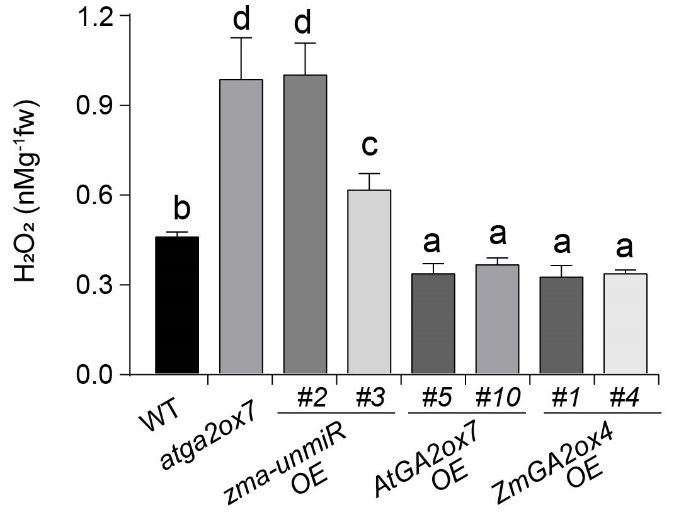


**Figure S8.** Investigation of H_2_O_2_ content in the inoculated leaves of indicated genotypes.

Healthy rosette leaves of 4-week-old plants were inoculated with 20 µl *Fusarium verticillioides* spore suspension, and then sampled at 5 days postinoculation for determination of H_2_O_2_ content. Data are means ± SD from three biological replicates. Letters above the bars indicate significant differences (*P* < 0.05 by Student’s *t* test).
