# Supplementary material for: A novel maize microRNA negatively regulates resistance to Fusarium verticillioides
Source: Mol Plant Pathol. 2022 Jun 14;23(10):1446–60. doi: 10.1111/mpp.13240 (PMC9452762; doi:10.1111/mpp.13240)
Supplement: Supplementary file 9 — Figure S9 Exogenous application of gibberellin (GA) alters rice resistance to Fusarium verticillioides. (a) Growth phenotype of KY131 rice seedlings. Fourteen‐day‐old seedlings were sprayed with H2O, GA (50 μM), or uniconazole (20 μM) once a day for 4 days and then photographed. Bar = 5 cm. (b, c) Disease symptoms of KY131 leaves upon F. verticillioides exposure. Bar = 1 cm. For panel (b), 14‐day‐old seedlings were treated as in panel (a) and then sprayed with F. verticillioides spore suspension (F. V) and sterile water (Mock) once a day for 6 days and photographed. For panel (c), 14‐day‐old seedlings were treated as in panel (a), and then the leaves were immersed in F. V spore suspension for 5 days and photographed [file MPP-23-1446-s003.docx]

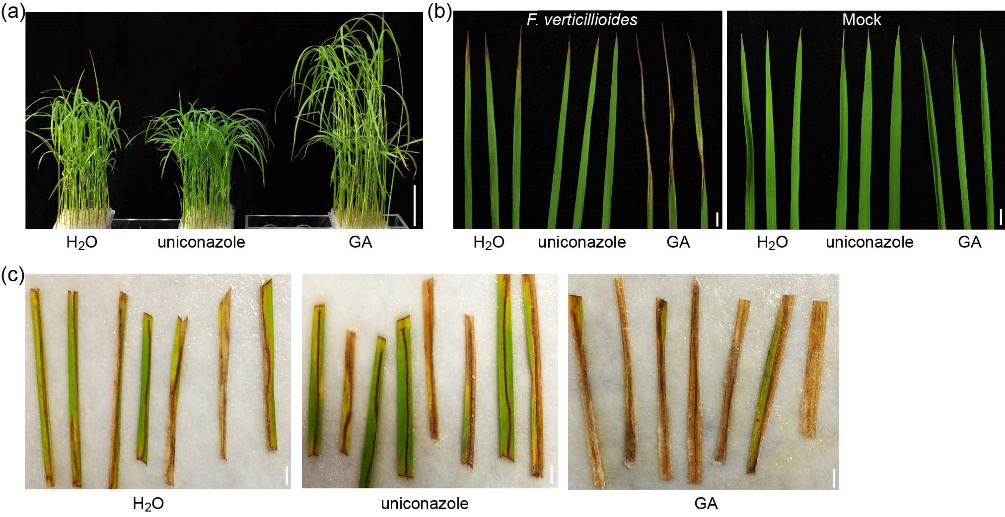


**Figure S9. Exogenous application of gibberellin (GA) alters rice resistance to *Fusarium verticillioides.***

(**a**) Growth phenotype of KY131 rice seedlings. 14-d-old seedlings were sprayed with H_2_O, GA (50 μM) or uniconazole (20 μM) once a day for 4 days, and then photographed. Bar = 5 cm. (**b, c**) The disease symptoms of KY131 leaves upon *F. verticillioides* exposure. Bar = 1 cm. For b, 14-day-old seedlings were treated as panel a, then sprayed with *F. verticillioides* spore suspension (*F. V*) and sterile water (Mock) once a day for 6 days and photographed. For c, 14-day-old seedlings were treated as panel a, then the leaves were immersed in *F. V* spore suspension for 5 days, then photographed.
