# Supplementary material for: A novel maize microRNA negatively regulates resistance to Fusarium verticillioides
Source: Mol Plant Pathol. 2022 Jun 14;23(10):1446–60. doi: 10.1111/mpp.13240 (PMC9452762; doi:10.1111/mpp.13240)
Supplement: Supplementary file 10 — Figure S10 Seedling growth difference and the promoter sequence divergence of zma‐unmiR4 between BT‐1 and N6. (a) Promoter sequence differences of zma‐unmiR4 between BT‐1 and N6 genotypes. Red letters indicate different bases. The red box indicates the zma‐unmiR4 sequence. (b) Phenotype difference between BT‐1 and N6 seedlings at 7 days after sowing (DAS) and 11 DAS. Bar = 7 cm [file MPP-23-1446-s004.docx]

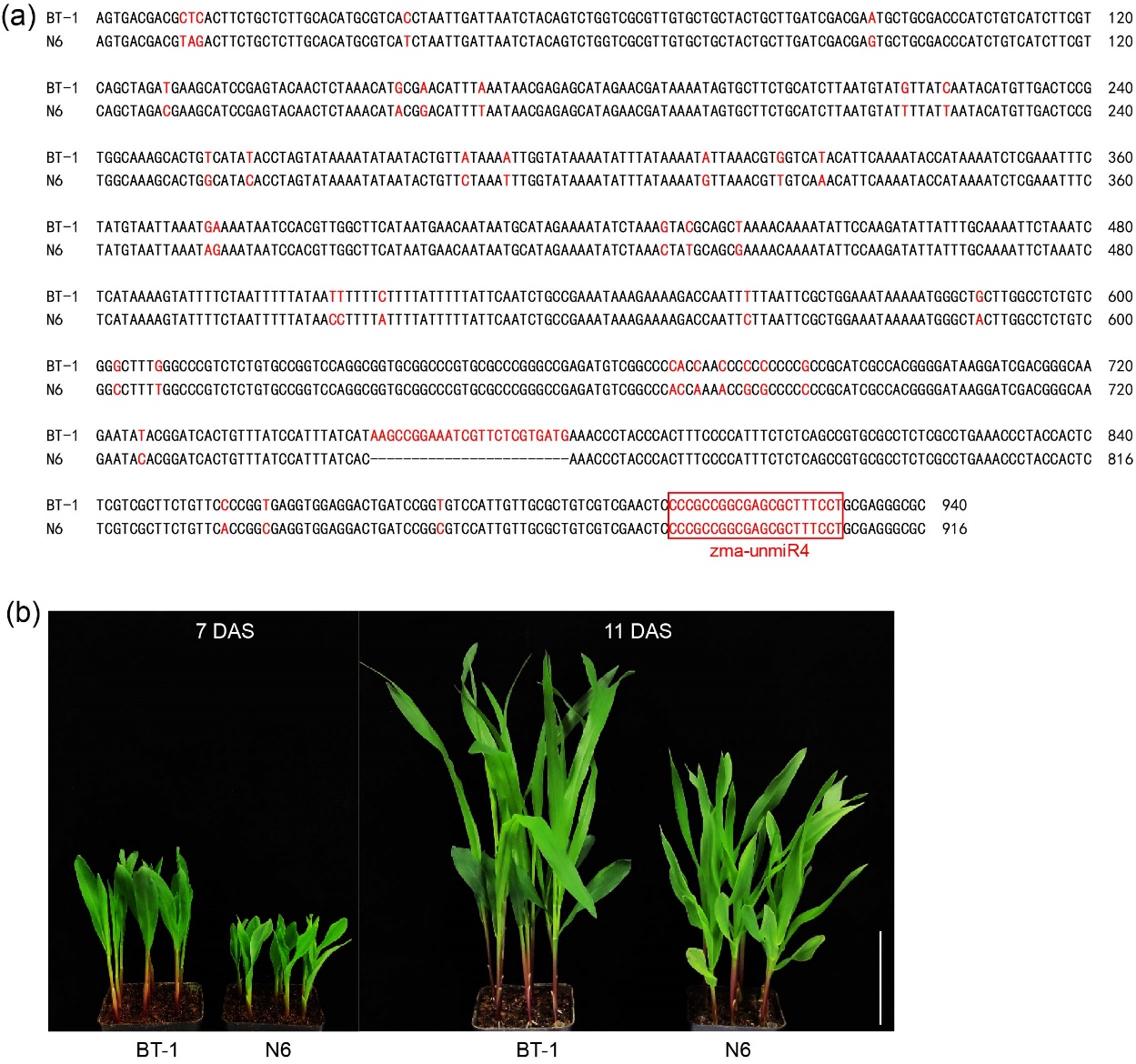


**Figure S10.** **Seedling growth difference and the promoter sequence divergence of zma-unmiR4 between BT-1 and N6.**

(a) Promoter sequence differences of zma-unmiR4 between BT-1 and N6 genotypes. Red letters indicate different bases. Red box indicates zma-unmiR4 sequence. (b) Phenotype difference between BT-1 and N6 seedlings at 7 days after sowing (DAS) and 11 DAS. Bar = 7 cm.
